# Supplementary material for: Selectivity of Lewy body protein interactions along the aggregation pathway of α-synuclein
Source: Commun Biol. 2021 Sep 23;4:1124. doi: 10.1038/s42003-021-02624-x (PMC8460662; doi:10.1038/s42003-021-02624-x)
Supplement: Supplementary file 2 — Supplementary Information [file 42003_2021_2624_MOESM2_ESM.pdf]

SUPPLEMENTARY INFORMATION

Selectivity of Lewy Body Protein Interactions along the Aggregation Pathway of α-Synuclein

André D. G. Leitão[1,2], Paulina Rudolffi-Soto[1], Alexandre Chappard[1,3], Akshay Bhumkar[1,4], Derrick Lau[1], Dominic J. B. Hunter[1,5], Yann Gambin[1,*], Emma Sierecki[1,*].

[1]EMBL Australia Node in Single Molecule Science and School of Medical Sciences, The University of New South Wales, Sydney, NSW 2031, Australia,

[2] Present Address: Department of Molecular Medicine, The Scripps Research Institute, La Jolla, California, CA 92037, United States of America.

[3] Present Address: School of Chemistry, The University of Edinburgh, Edinburgh, EH9 3FJ, United Kingdom.

[4] Present Address: Woolcock Institute of Medical Research, University of Sydney, Sydney, NSW 2037, Australia.

[5]Institute for Molecular Bioscience, The University of Queensland, Brisbane, QLD 4072, Australia.

[*] To whom correspondence should be addressed: Yann Gambin, y.gambin@unsw.edu.au and to Emma Sierecki, e.sierecki@unsw.edu.au.

Supplementary method:

Alphascreen assay:

Direct interactions between monomeric N-terminal mCherry α-SYN and the N-GFP Lewy Body proteins were assessed using AlphaScreen. AlphaScreen is a nanobead assay, where the two interacting proteins bring a “donor” bead and an “acceptor” bead in close proximity. The donor bead is coated with streptavidin, which binds biotin-coupled GFP nanotrap. The GFP nanotrap recruits the GFP-tagged protein (LB protein). The acceptor bead is coated with anti-myc antibody that binds to the mCherry-myc tag of α-SYN. The donor bead contains phtalocyanine, a photosensitizer that converts ambient oxygen to an excited state upon illumination at 680 nm. The resulting singlet oxygen has a half life of 4µs, in which it can diffuse ~200 nm in solution, before returning to its triplet unexcited state. If the two proteins (N-Cherry α-SYN and N-GFP LB protein) interact, the beads are brought within that distance and the singlet oxygen reacts with thioxene derivatives in the acceptor bead, leading to emission of light at 520-620 nm. The technique has high sensitivity, thanks to the local increase of the concentration of the proteins at the surface of the beads, allowing the formation of low affinity complexes. However, AlphaScreen signal is dependent on the concentration of the protein: at optimal protein-to-bead ratios (when the beads are fully coated), a maximum signal is detected as shown by a typical ‘hook effect’ in the quantified luminescence emitted (**Figure 2-b**).

In this study we take advantage of another aspect of the system. We performed 4 serial dilutions of the samples containing the co-expressed proteins to find the optimal proteins-to-beads ratio. Typically, optimal coating of the beads is achieved when we dilute the LTE by 3-4 orders of magnitude. At these (lower) concentrations, dissociation rates are high and even aggregation-prone proteins will be largely monomeric. Therefore, the presence of few aggregates should not overwhelm the system and interactions between aggregates will have the same influence as interactions between two monomers.

Two-color Coincidence Detection (TCCD) to study PPIs:

Two-color coincidence detection (TCCD) refers to the simultaneous excitation of two fluorophores by two independent lasers and detection of the fluorescence emitted as a result. TCCD relies on the diffusion of a dual-labelled complex (such as an heterogenous aggregate of two differently tagged species) through the confocal volume, which gives rise to coincident bursts of fluorescence on both channels. This approach can be used to sensitively detect the presence of dual-labelled molecules, even in the presence of a large excess of singly labelled molecules, as are typically our cell-free expressed samples of fluorescent proteins. It can also be used to determine the stoichiometry of the complex (hence, of the interaction).

The identification of fluorescent events in TCCD is achieved by counting the number of photons emitted at a set interval time τ. First, however, one must define what is to be considered an ‘event’. This is achieved by establishing thresholds, above which bursts are recorded. The presence of two events on both channels at the same bin-time (selected by the operator) is read as a coincident event. The simple binning-threshold method for event selection functions extremely well providing that signal

to noise ratio is sufficiently high, and the interval time is longer than the diffusion time of the molecule across the probe volume.

TCCD is now being applied to increasingly complex systems, bringing about issues in the detection of fluorescent events. Systems where the expression level of proteins is difficult to control or where background levels are too high may affect the threshold selection. Another factor we had to overcome in this study is the occurrence of chance coincident events, i.e. when two species tagged with different colours enter the confocal volume at the same time by chance. Ideally, chance coincident events should represent only a fraction of the total number of events, but sometimes chance for co-diffusion can increase. This is particularly critical in our studies of interactions with α-SYN fibrils, which were performed at single molecule concentrations, where dissociation rates can be higher.

To tackle these limitations the choice of threshold is critical, and here we followed an optimized methodology published by Clarke and colleagues[13], as described next.

Association Quotient (Q)

Choosing optimal thresholds to define an 'event' is a critical step in burst detection: the threshold should be high enough that exceeds the background level and minimizes detection of chance co-diffusions; it should also be low enough that ensures collection of enough fluorescent events.

Correction for chance can be incorporated into the calculation of an association quotient, Q, that defines a fraction of coincident events from the total number of events. Clarke *et al*[13] have demonstrated recently that appropriate thresholds can be calculated automatically by plotting the population of Q values as a function of the thresholds and finding the maximum Q values. According to the authors, Q is defined as:

$$Q = \frac{(C - E)}{(A + B - (C - E))} \qquad (1)$$

Where *A* and *B* are the events in the two channels, the observed rate of coincident events is C and the estimated rate of events that occur by chance is given by *E*, which in turn can be defined as:

$$E = AB\tau \qquad (2)$$

Where A and B are the total rates of events in the two channels and τ is the interval time in seconds. In **Figure 3-a-iii** we show a simple representation of a Q value calculated for a control co-expression of C-GFP-tagged G51D α-SYN with C-Cherry-tagged G51D. We used the approach described in the manuscript mentioned above[13] to calculate, for each time trace of co-expressed m-Cherry- and GFP-tagged proteins the optimal threshold, as shown in **Figure S3**.

In sum, by plotting thresholds against each corresponding Q value, we obtain a color-field graph such as represented in **Figure S3**, and we are then able to define the optimal threshold as the lower threshold that gives the maximum Q value. This is represented in our example in **Figure S3 (panels B and D)** as the darker warm colours and in the raw trace (**panels A and C**) as the blue lines. Below

this threshold, the background is too high, increasing the chance (E) for non-interacting partners to co-diffuse (therefore decreasing Q in equation 5); on the other hand, above this threshold we sample a number of events that is too low for a representative analysis. In the color-field maps above, we see high Q values at the top of the graph as well, owing to the very few events that are detected at such high levels, which will mostly be picked as coincident; hence we automatically select the lower threshold. This example shows the importance of threshold selection in our work, as we performed hundreds of coexpressions of proteins. Therefore, a fast, expedite and automated way of calculating association quotients in a high throughput manner was required.

By applying this methodology to all our co-expression traces we obtained consistent Q values, as shown by relatively low dispersity of replicates in our results section.

<u>Coincidence ratio for stoichiometry analysis</u>

In this work we calculated stoichiometries of interactions for identified binders of α-SYN in order to determine the relative presence of each species in heterogenous aggregates. We collected the population of coincident events (using the same threshold selection method as described above) and calculated a coincidence ratio C that gives the average fraction of mCherry fluorophores in the aggregate, as defined by the formula below:

$$\mathrm{C} = \frac{\mathrm{I_{GFP}}}{(\mathrm{I_{Cherry}} + \mathrm{I_{GFP}})} \qquad (3)$$

Where I_{Cherry} and I_{GFP} are the threshold-subtracted intensity in the Cherry and GFP channels, respectively. It should be noted that before applying this fraction for each event, the intensities of the GFP and Cherry bursts were corrected for background and leakage (6% leakage of the GFP intensity into the Cherry channel). By applying equation 3 to our population of coincident events we obtain a number between 0 (absence of GFP fluorescence) and 1 (absence of Cherry fluorescence). Histograms of single-molecule coincidence were obtained by measuring >400 events per interaction.

SUPPLEMENTARY FIGURE 1:

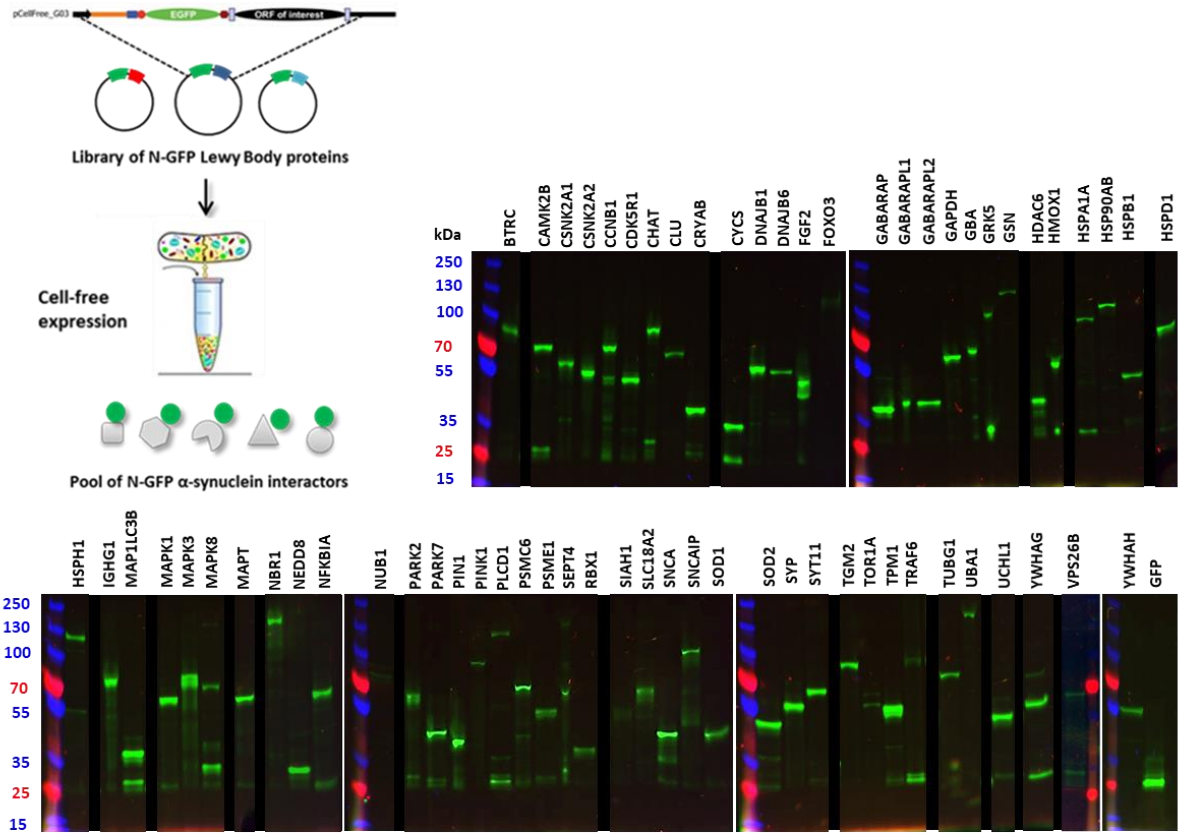

Figure S1. SDS-PAGE gels of Lewy Body proteins upon cell-free expression. An library of 65 constructs was cloned using Gateway cloning vectors and obtained DNA constructs were expressed in the cell-free reaction of Leishmania tarentolae extract (LTE). Gels were imaged using ChemiDoc MP imaging system with Image Lab software. Expression was allowed to occur for 2h at 27C and products were loaded on SDS-PAGE gels for quality control of protein expression. Lanes from the same gel were rearranged and are presented here in alphabetical order; rearrangement of lanes is indicated by a black vertical line and a wider space separating each lane. Proteins showing a band at the expected size and with minimal truncation profile were used throughout this study. For full library and sizes of LB proteins see **Table S1**. This supplement figure refers to **Figure 1**.

SUPPLEMENTARY TABLE 1:

GENE	NAME	UNIPROT ID	LENGTH (aa)	MW (Da)	MW + GFP (Da)	Family keyword	Function keyword
BTRC	F-box/WD repeat-containing protein 1A	Q9Y297	605	68,867	95,753	Ubiquitin	Degradation
CAMK2B	Calcium/calmodulin-dependent protein kinase type II subunit beta	Q13554	666	72,678	99,564	Kinase	Brain
CCNB1	G2/mitotic-specific cyclin-B1	P14635	433	48,337	75,223	Mitosis	Cell cycle
CDK5R1	Cyclin-dependent kinase 5 activator	Q8N619	307	34,060	60,946	Kinase	Brain
CHAT	Choline O-acetyltransferase	P28329	748	82,536	109,422	Enzyme	Brain
CLU	Clusterin	P10909	449	52,495	79,381	Chaperone	Folding
CRYAB	Alpha-crystallin B chain	P02511	175	20,159	47,045	Chaperone	Folding
CSNK2A1	Casein kinase II subunit alpha	P68400	391	45,144	72,030	Kinase	Enzyme
CSNK2A2	Casein kinase II subunit alpha'	P19784	350	41,213	68,099	Kinase	Enzyme
CYC5	Cytochrome c	P99999	105	11,749	38,635	Cytochrome	Enzyme
DNAJB1	DnaJ homolog subfamily B member 11	Q9UBS4	358	40,514	67,400	Chaperone	Folding
DNAJB6	DnaJ homolog subfamily B member 6	O75190	326	36,087	62,973	Chaperone	Folding
FGF2	Fibroblast growth factor 2	P09038	288	30,770	57,656	Growth Factor	Apoptosis
FOXO3	Forkhead box protein O3	O43524	673	71,277	98,163	Transcription Factor	Gene Regulation
GABARAP	GABA(A) receptor-associated protein	Q6IAW1	117	13,918	40,804	Autophagosome	Degradation
GABARAPL1	Gamma-aminobutyric acid receptor-associated protein-like 1	Q9H0R8	117	14,044	40,930	Autophagosome	Degradation
GABARAPL2	Gamma-aminobutyric acid receptor-associated protein-like 2	P60520	117	13,667	40,553	Autophagosome	Degradation
GAPDH	Glyceraldehyde-3-phosphate dehydrogenase	P04406	335	36,053	62,939	Enzyme	Gene Regulation
GBA	Lysosomal acid glucosylceramidase	P04062	536	59,716	86,602	Enzyme	Brain
GFP	Green fluorescent protein	P42212	238	26,886	53,772		
GRK5	G protein-coupled receptor kinase 5	P34947	590	67,787	94,673	Kinase	Enzyme
GSN	Gelsolin	P06396	782	85,698	112,584	Actin binding	Brain
HDAC6	HDAC6 protein	Q9BRX7	146	16,399	43,285	Histone	Gene Regulation
HMOX1	Heme oxygenase	Q96DI8	288	32,800	59,686	Enzyme	Blood vessel
HSP90AB	Heat shock protein HSP 90-beta	P08238	724	83,264	110,150	Chaperone	Folding
HSPA1A	Heat shock 70 kDa protein 1A	P0DMV8	641	70,052	96,938	Chaperone	Folding
HSPB1	Heat shock protein beta-1	P04792	205	22,783	49,669	Chaperone	Folding
HSPD1	60 kDa heat shock protein, mitochondrial	P10809	573	61,055	87,941	Chaperone	Folding
HSPH1	Heat shock protein 105 kDa	Q92598	858	96,865	123,751	Chaperone	Folding
IGHG1	Immunoglobulin heavy constant gamma 1	P01857	330	36,106	62,992	glycoprotein	Immunity
MAP1LC3B	Microtubule-associated proteins 1A/1B light chain 3B	Q9GZQ8	125	14,688	41,574	cytoskeleton	Brain
MAPK1	Mitogen-activated protein kinase 1	P28482	360	41,390	68,276	kinase	Enzyme
MAPK3	Mitogen-activated protein kinase 3	P27361	379	43,136	70,022	kinase	Enzyme
MAPK8	Mitogen-activated protein kinase 8	A1L4K2	427	48,088	74,974	kinase	Inflammation
MAPT	Microtubule-associated protein tau (amylodogenic isoform 2N4R)	P10636-8	758	45,850	72,736	aggregate	Brain
NBR1	Next to BRCA1 gene 1 protein	Q14596	966	107,413	134,299	autophagosome	Brain
NEDD8	neural precursor cell expressed, developmentally down-regulated 8	Q15843	81	9,072	35,958	ubiquitin	Degradation
NFKB1A	NF-kappa-B inhibitor alpha	P25963	317	35,609	62,495	transcription factor	Inflammation
NUB1	NEDD8 ultimate buster 1	Q9Y5A7	615	70,538	97,424	ubiquitin	Degradation
PARK2	E3 ubiquitin-protein ligase parkin	O60260	465	51,641	78,527	ubiquitin	Brain
PARK7	Protein/nucleic acid deglycase DJ-1	Q99497	189	19,891	46,777	enzyme	Brain
PIN1	Peptidyl-prolyl cis-trans isomerase NIMA-interacting 1	Q13526	163	18,243	45,129	isomerase	Enzyme
PINK1	Serine/threonine-protein kinase PINK1, mitochondrial	Q9BXM7	581	62,769	89,655	kinase	Brain
PLCD1	1-phosphatidylinositol 4,5-bisphosphate phosphodiesterase delta-1	P51178	756	85,665	112,551	lipase	Membrane
PSMC6	26S proteasome regulatory subunit 10B	P62333	389	44,173	71,059	proteasome	Degradation
PSME1	Proteasome activator complex subunit 1	Q06323	249	28,723	55,609	proteasome	Degradation
RBX1	E3 ubiquitin-protein ligase RBX1	P62877	108	12,274	39,160	ubiquitin	Gene regulation
SEPT4	Septin-4	O43236	478	55,098	81,984	cytoskeleton	Brain
SIAH1	E3 ubiquitin-protein ligase SIAH1	Q8IUQ4	282	31,123	58,009	ubiquitin	Brain
SLC18A2	Synaptic vesicular amine transporter	Q05940	514	55,713	82,599	transport	Brain
SNCA	Alpha-synuclein	P37840	140	14,460	41,346	membrane association	Brain
SNCAIP	Synphilin-1	Q9Y6H5	919	100,409	127,295	interacting partner	Brain
SOD1	Superoxide dismutase [Cu-Zn]	P00441	154	15,936	42,822	enzyme	Brain
SOD2	SOD2 protein	Q96AM7	140	15,745	42,631	enzyme	Brain
SYP	Synaptophysin	P08247	313	33,845	60,731	membrane association	Brain
SYT11	Synaptotagmin-11	Q9BT88	431	48,297	75,183	membrane association	Brain
TGM2	Protein-glutamine gamma-glutamyltransferase 2	P21980	687	77,329	104,215	enzyme	Brain
TOR1A	Torsin-1A	O14656	332	37,809	64,695	enzyme	Brain
TPM1	Tropomyosin alpha-1 chain	P09493	284	32,709	59,595	cytoskeleton	Brain
TRAF6	TNF receptor-associated factor 6	Q9Y4K3	522	59,573	86,459	adaptor	Inflammation
TUBG1	Tubulin gamma-1 chain	P23258	451	51,170	78,056	microtubule	Cytoskeleton
UBA1	Ubiquitin-like modifier-activating enzyme 1	P22314	1,058	117,849	144,735	ubiquitin	Degradation
UCHL1	Ubiquitin carboxyl-terminal hydrolase isozyme L1	P09936	223	24,824	51,710	ubiquitin	Degradation
VPS26B	Vacuolar protein sorting-associated protein 26B	Q4G0F5	336	39,155	66,041	retromer	Protein transport
YWHAG	14-3-3 protein gamma	P61981	247	28,303	55,189	scaffold	Signalling hubs
YWHAH	14-3-3 protein eta	Q04917	246	28,219	55,105	scaffold	Signalling hubs

Table S1. LB proteins that were cloned and used for cell-free expression in this study. Proteins are organized alphabetically from gene name. Length is represented in aminoacids (aa). Molecular weight in daltons (Da). Expected sizes of GFP-tagged constructs are represented in column 'MW+GFP'. Family and function keywords were acquired from Panther Gene Onthology software. This supplementary table refers to **Figure 1** and **Figure S1**.

SUPPLEMENTARY FIGURE 2:

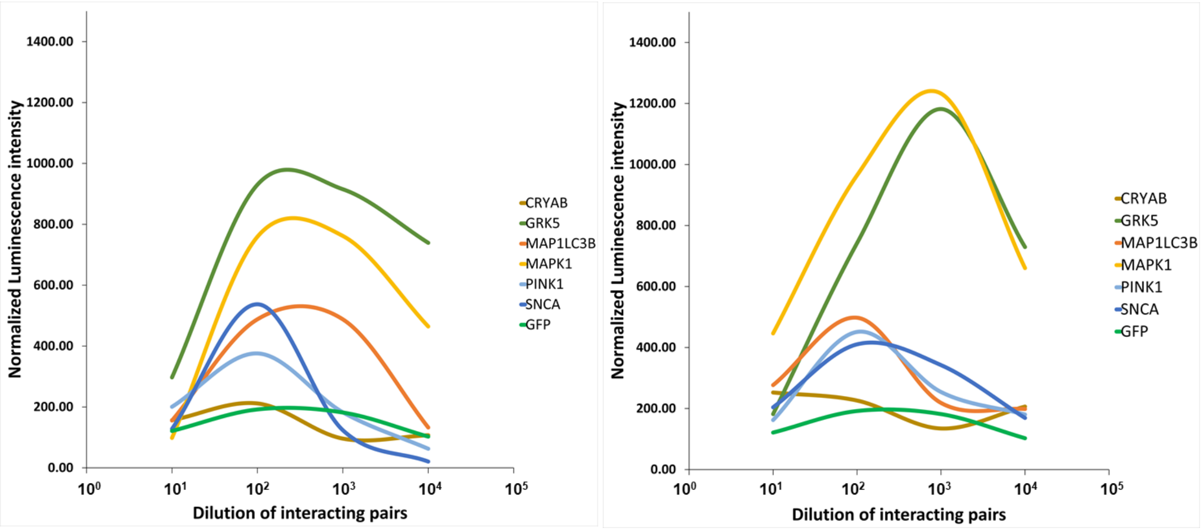

Figure S2. ALPHAScreen Luminescence curves for cell-free-expressed interacting pairs. Left and right panels represent two different biological replicates, each depicting average curves of 4 measurements. 7 Different pairwise interactions with α-SYN are shown: 5 GFP-tagged Lewy Body proteins, α-SYN itself and GFP alone. This supplementary figure refers to **Figure 2.**

SUPPLEMENTARY FIGURE 3:

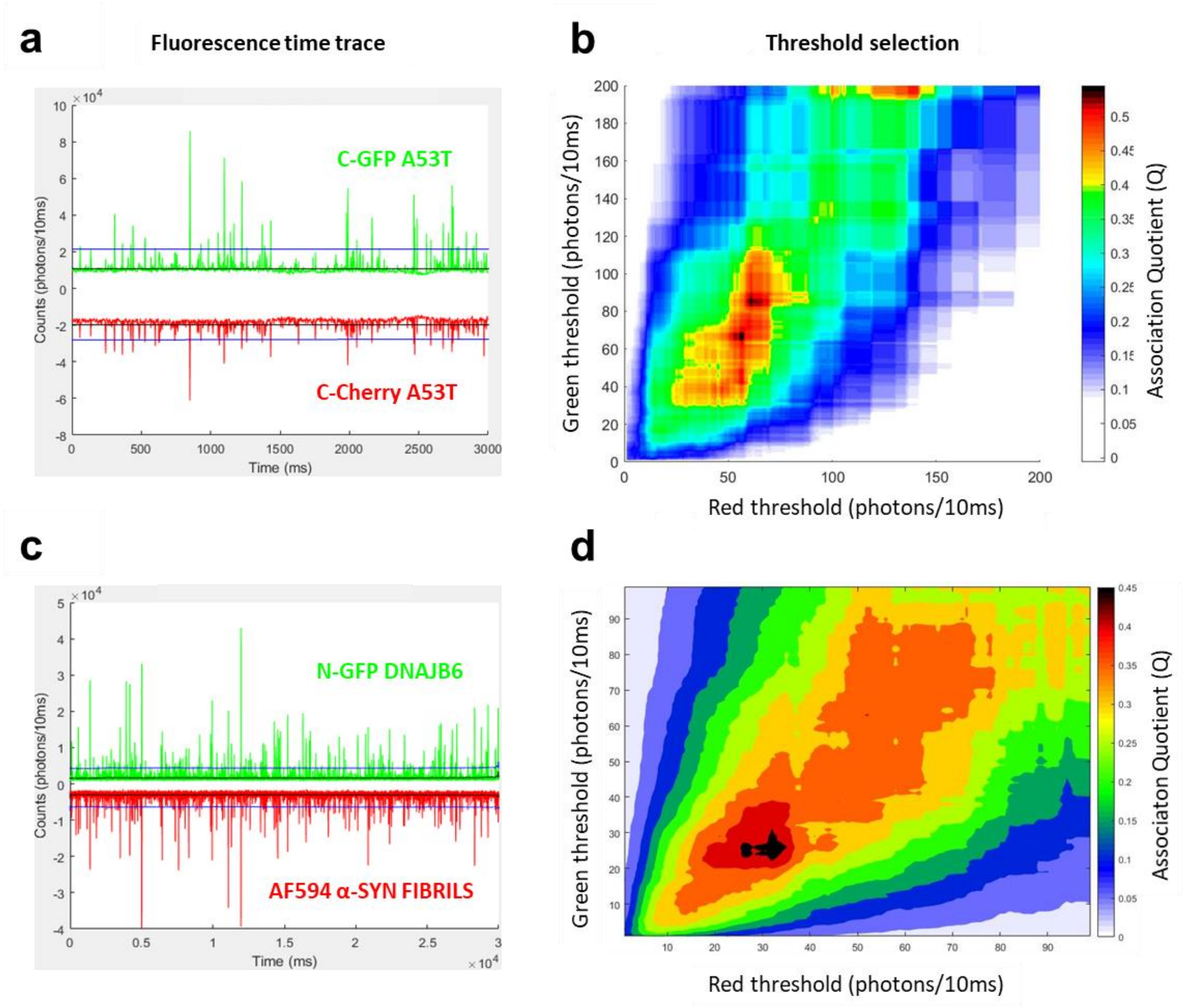

Figure S3. Optimal threshold selection for calculation of association quotients. a Fluorescence time trace for coexpression of C-GFP A53T and C-Cherry A53T synucleins. Black line (lower) is average intensity; blue line (upper) is the optimal threshold as shown from maximum Q in B. For calculation of threshold and Q, data was binned to 10 ms. **b** Color-field map for threshold selection for the trace on the left panel. Optimal thresholds are the ones which give the maximum Q values. Color-field map was acquired by normalizing the binned data from the raw trace to the background. Thresholds were calculated by binning the data to 10ms, in order to filter out small bursts that only cross the threshold for short periods of time (i.e. bursts that cross the threshold for less than 10ms were disregarded). **c,d** The same procedure was applied to single-molecule traces of binding to AlexaFluor594-labelled α-SYN fibrils. Data was binned and normalized in a similar manner. This supplementary figure refers to **Figures 3 and 5.**

SUPPLEMENTARY FIGURE 4:

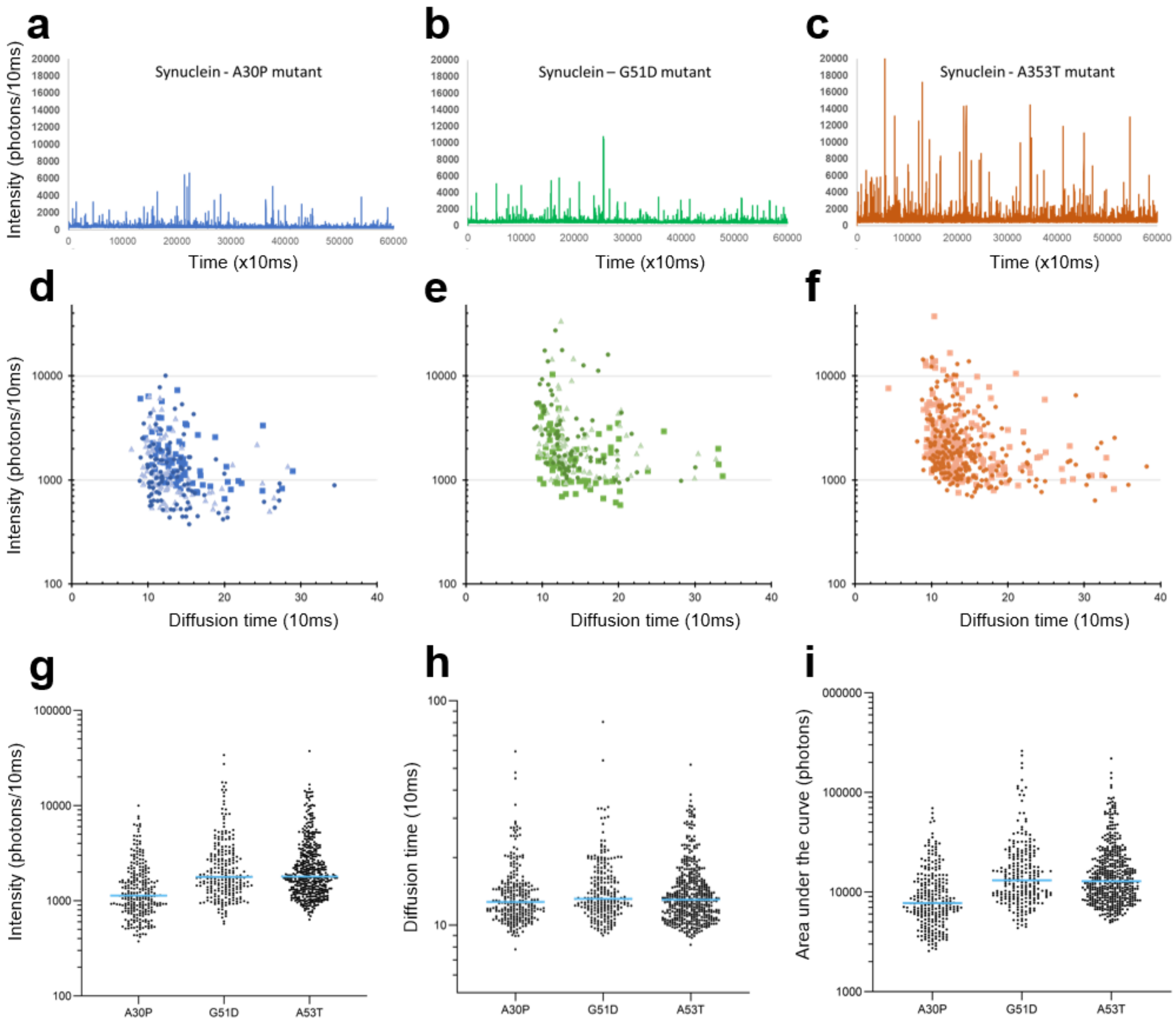

Figure S4: Fingerprinting of the three Synuclein mutants expressed in cell-free.

A30P, G51D and A53T α-SYN mutants were expressed in our cell-free system with a C-terminal GFP tag and the size of the aggregates was characterized using a dedicated 3D printed microscope[90]. On this instrument, each Synuclein aggregate can be analysed for its residence time in the focal volume, using a recently developed algorithm[91]. **a-c** Raw fluorescence trace obtained for the three mutants on the AttoBright setup; the binning time of the fluorescence intensity is 10ms. **d-f** Analysis of diffusion time (residence time in the focal volume) and maximal intensity of the peak for the three mutants. The difference colours correspond to replicates. **g-i** Violin plots of the maximal Intensity, diffusion times and area under the curve for the three mutants.

SUPPLEMENTARY FIGURE 5:

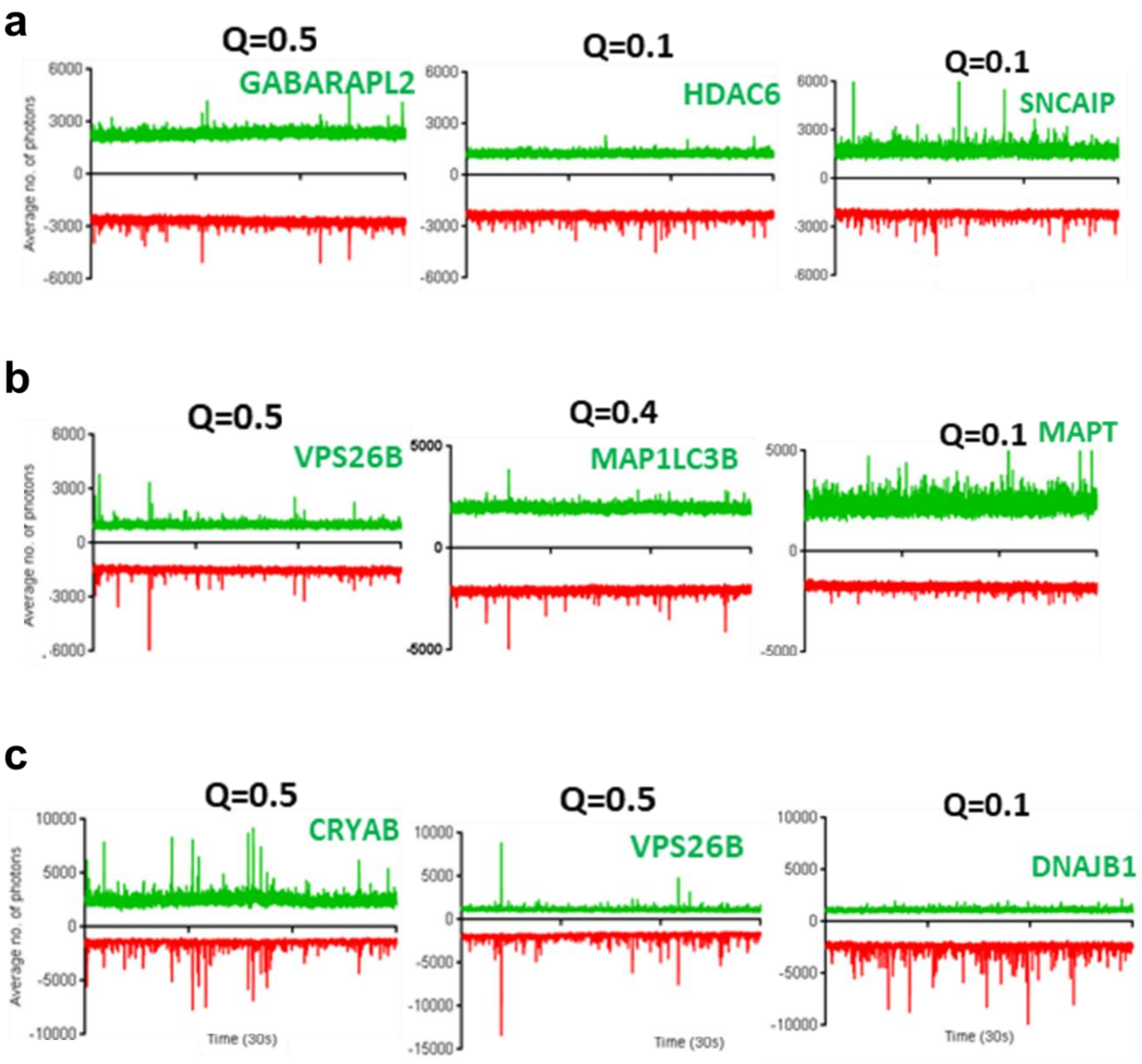

Figure S5. Example of time traces for cell-free co-expressions between mutant synucleins and Lewy Body proteins. Fluorescence time trace for coexpression of C-mCherry synuclein and C-GFP Lewy Body proteins. **a,b,c** Coexpressions with A30P, G51D and A53T, respectively. Insets represent a single replicate of a 30 second time trace for each co-expression. A total of 4 replicates were obtained and averaged to obtain an average Association Quotient (Q) which was normalized to obtain Binding Indexes throughout this study. This supplementary figure refers to **Figure 3**.

SUPPLEMENTARY FIGURE 6:

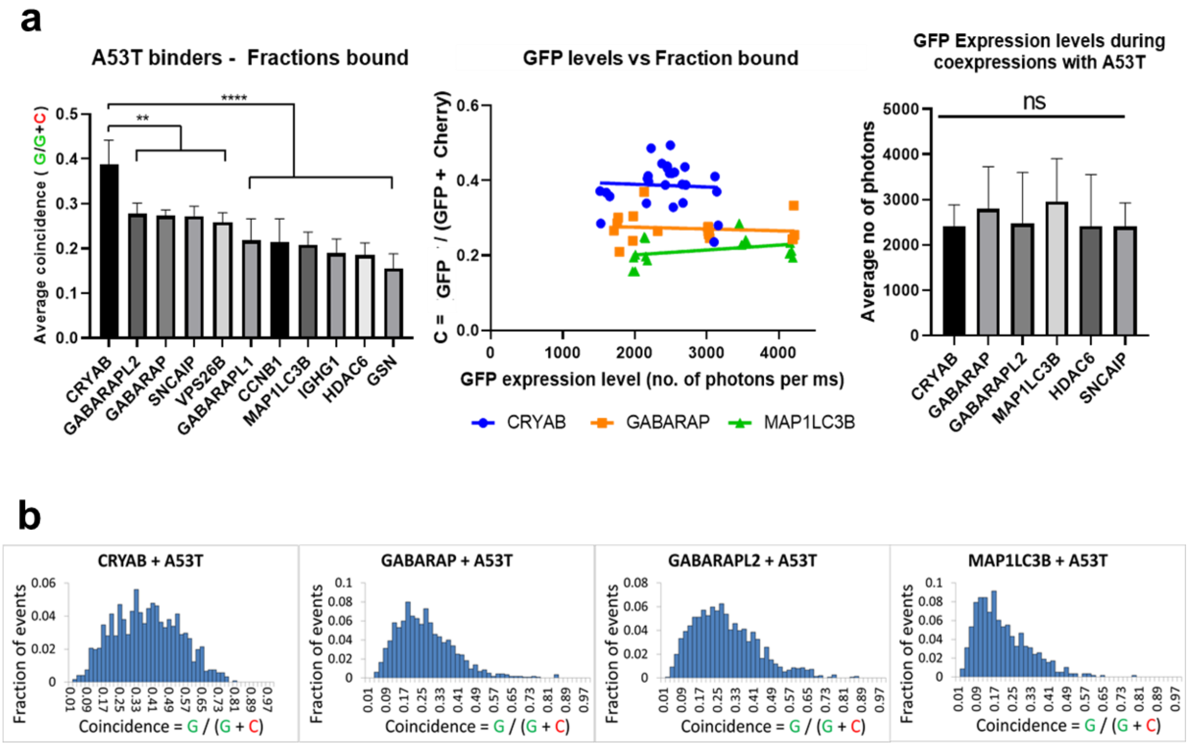

Figure S6. Analysis of bound fractions for binders of A53T α-SYN conformers. a The variation of GFP expression levels is negligible between each coexpression replicate (middle graph) and all coexpressions were performed at similar GFP expression levels between different proteins (right bar graph). Stoichiometries (left) were measured as the fraction of Cherry-tagged species in coincident events (C=C/(C+G)). **** p≤0.0001, ** p≤0.01 (n=3). **b** Distributions of apparent stoichiometries across all the coincident events are depicted. Error bars show mean±SEM. This supplementary figure refers to **Figure 3**.

SUPPLEMENTARY FIGURE 7:

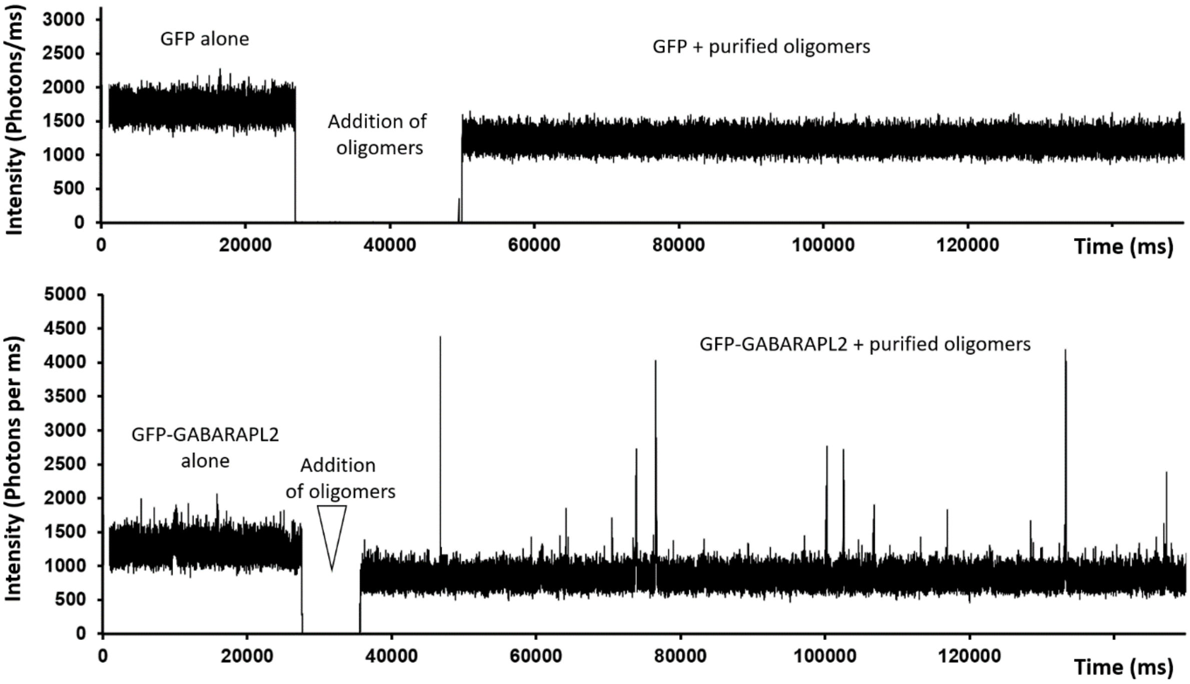

Figure S7. Kinetics of binding of GABARAPL2 to purified oligomers

(top): Raw trace of fluorescence measured for monomeric GFP expressed in cell-free, diluted in AlphaScreen buffer. The protein was measured diluted in buffer, then recording was blocked during addition and mixing of the purified oligomers. The last part of the measurement corresponds to GFP in the presence of oligomers. The GFP protein remains monomeric and B values remain unchanged (see **Figure 4**).

(bottom): raw fluorescence trace obtained for GABARAPL2 expressed in cell-free with a GFP tag at the N-terminus, before and after addition of purified oligomers. Brighter peaks appear only after addition of the oligomers, showing that multiple GABARAPL2 are recruited on the same particle and co-diffuse. This binding causes an increase in the Brightness values, as shown in **Figure 4**. Note that the Brightness value is concentration-independent in the range of intensities used here (between 400 photons/ms and 5000 photons/ms)[92, 93].

SUPPLEMENTARY FIGURE 8:

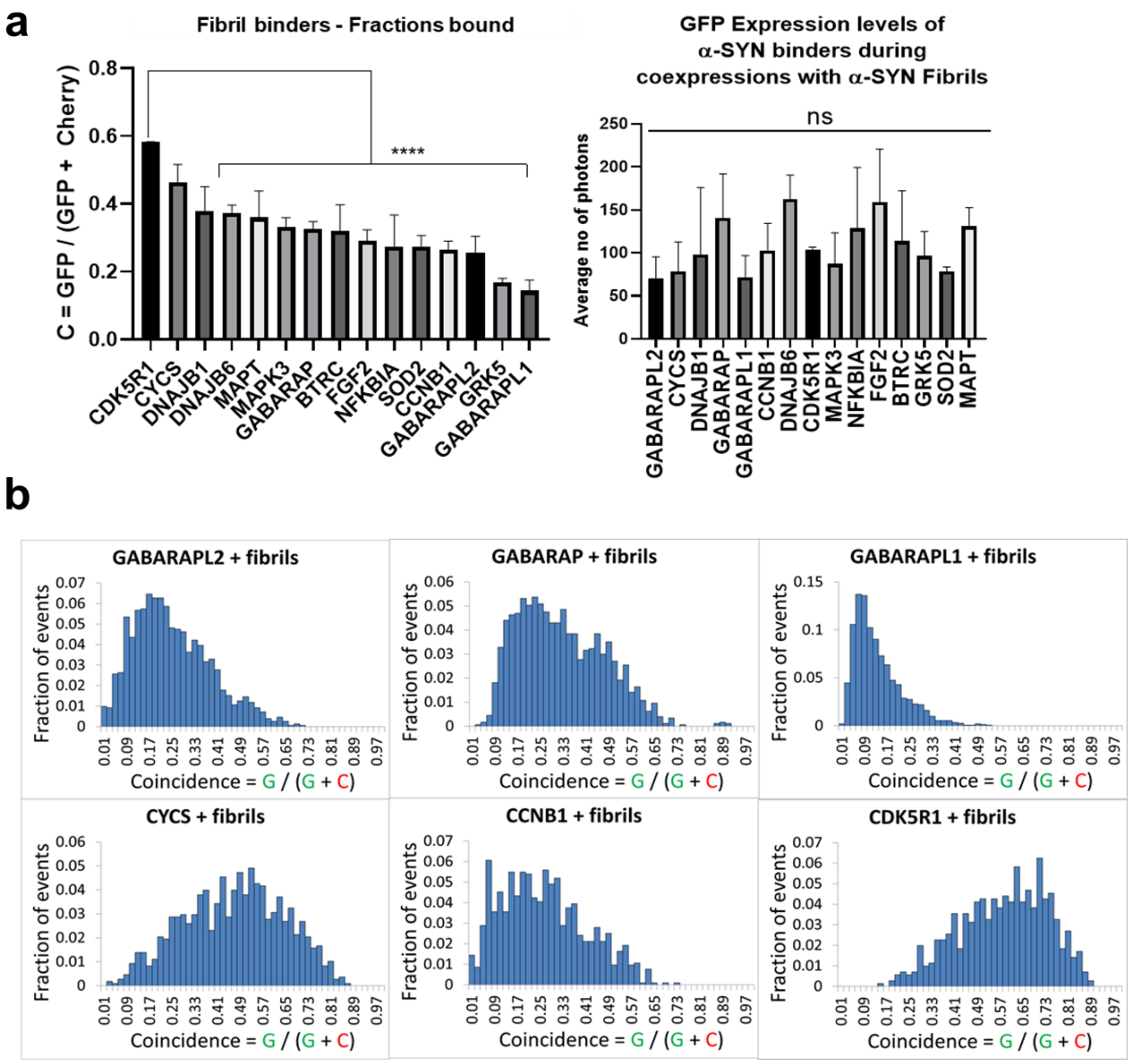

Figure S8. Analysis of bound fractions for interactions with fibrils of α-SYN. a Differences in bound fractions are shown across the 17 binders with the highest Q values (left graph). GFP expression levels did not vary significantly across all the binders tested (right graph). **b** Distributions of coincidence ratios as defined by the relative presence of Cherry species in heterogeneous aggregates. Values represent apparent stoichiometry since these do not account for the 10% incorporation rate of AF594 during labelling. *** $p \leq 0.001$, $n=3$. Error bars show mean±SEM. This supplementary figure refers to **Figure 5**.

SUPPLEMENTARY FIGURE 9:

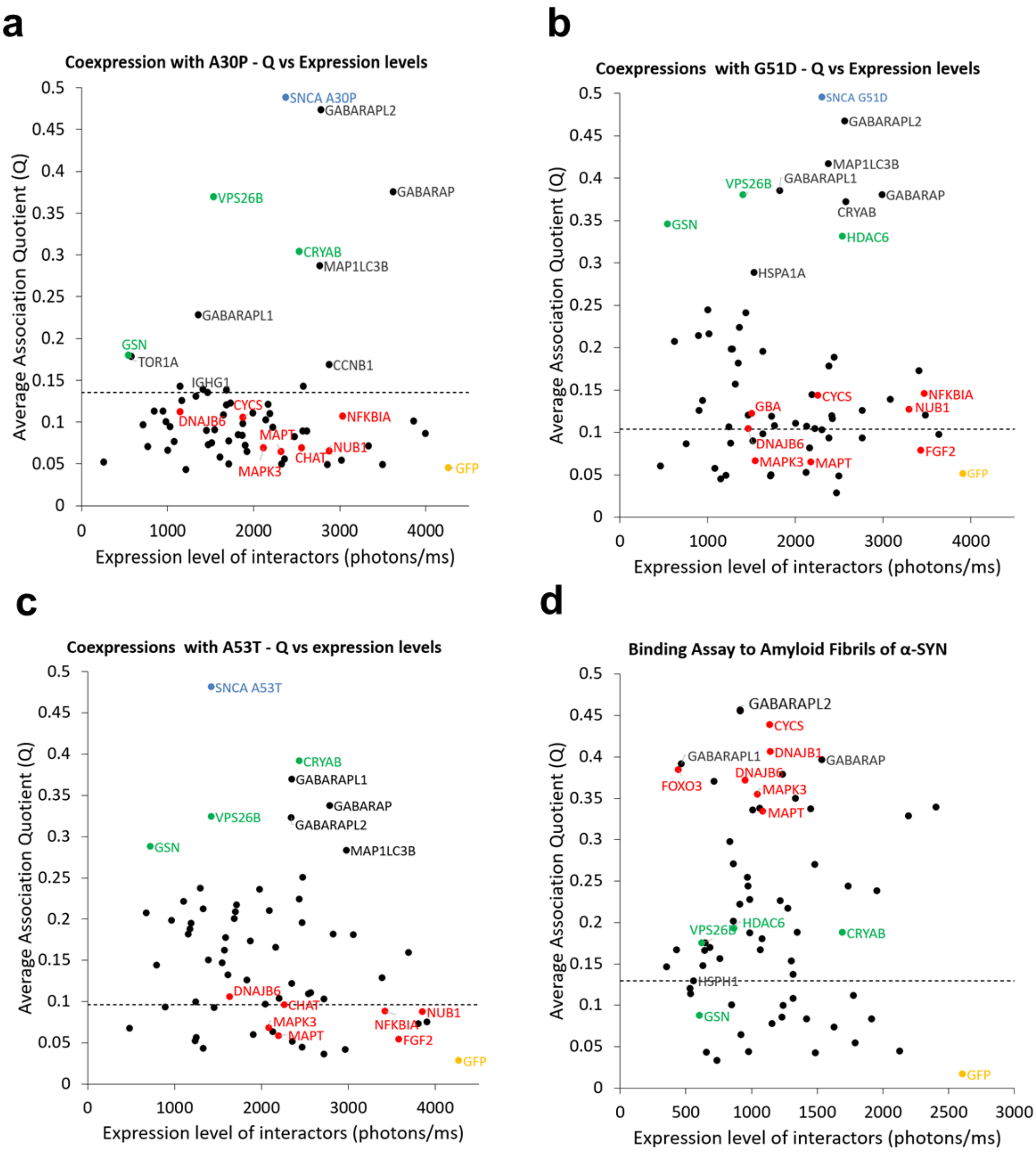

Figure S9. Effect of concentrations on association quotients with aggregated forms of alpha-synuclein. Plots of average Q values and expression levels of each interactor. Red – interactors with highest Q values with fibrils; Green – interactors with highest Q values for coexpression with oligomeric alpha-synuclein; blue – positive control; yellow – negative control (GFP alone). Dashed line represents the threshold above which Q values are statistically different from Q value of negative control (GFP). **(a, b** and **c)** Plots for cell-free coexpression of oligomeric alpha-synuclein and Lb partners; **d** expression of LB partners in the presence of pre-formed recombinant fibrils of α-SYN.

SUPPLEMENTARY FIGURE 10:

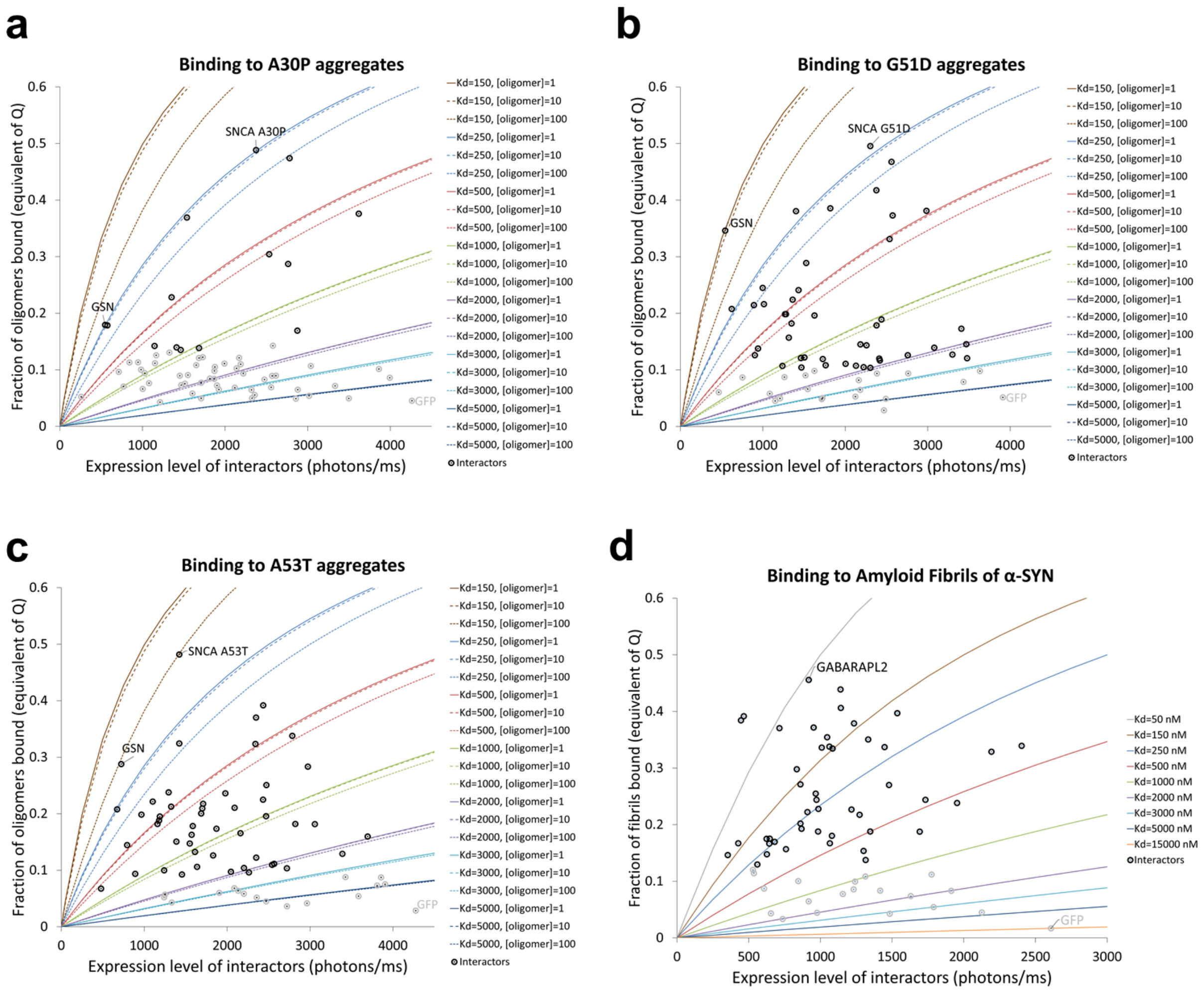

Figure S10. Simulation of binding curves for interactions with aggregated forms of α-Synuclein. Plots of Association Quotients versus Expression levels of interactors were merged with simulation curves for binding affinities. Binding curves were simulated for various conditions of dissociation constants (Kd) and concentrations of aggregated species. For the concentration of oligomers (in **a, b** and **c**) we simulated a variation between 1 and 100 nM of monomer equivalent, whereas for fibrils (in **d**) the concentration was pre-determined at 1 μM, during the purification and labelling of synuclein. Q values are here assumed as equivalent to fraction of interactors bound to aggregates, since Q represents the number of coincident events, from the total number of events. Average Q values of 3 independent results for each interaction are represented. Interactors (black circles) are defined as having Q values significantly higher than the Q value of the GFP monomer; non-interactors are shown as gray circles.

Supplementary Note (Figure S10)

This work is a high throughput study of over 60 interactors and, as such, is not designed to calculate absolute affinities but rather focuses on selectivity of interactions. For simplicity, we will assume that the Association Quotient Q can be considered an estimation of the fraction of proteins bound or co-aggregated. We created simulations of various apparent affinities in Q vs expression levels plots (Figure 7). The fractions of bound species were calculated based on the following formula of the dissociation constant for a first-order interaction:

$$\mathrm{Kd} = [\mathrm{oligomer/fibril}] \times (\mathrm{fraction\ bound} - 1) + [\mathrm{protein}] \times \left(\frac{1}{\mathrm{fraction\ bound}} - 1\right) \qquad (4)$$

It should be noted that this exercise assumes: i) that each aggregate of synuclein is one binding entity to which the rules of monomeric binding apply and ii) that Q directly informs on fraction bound (since it is the number of coincident events from the total population of events).

SUPPLEMENTARY FIGURE 11:

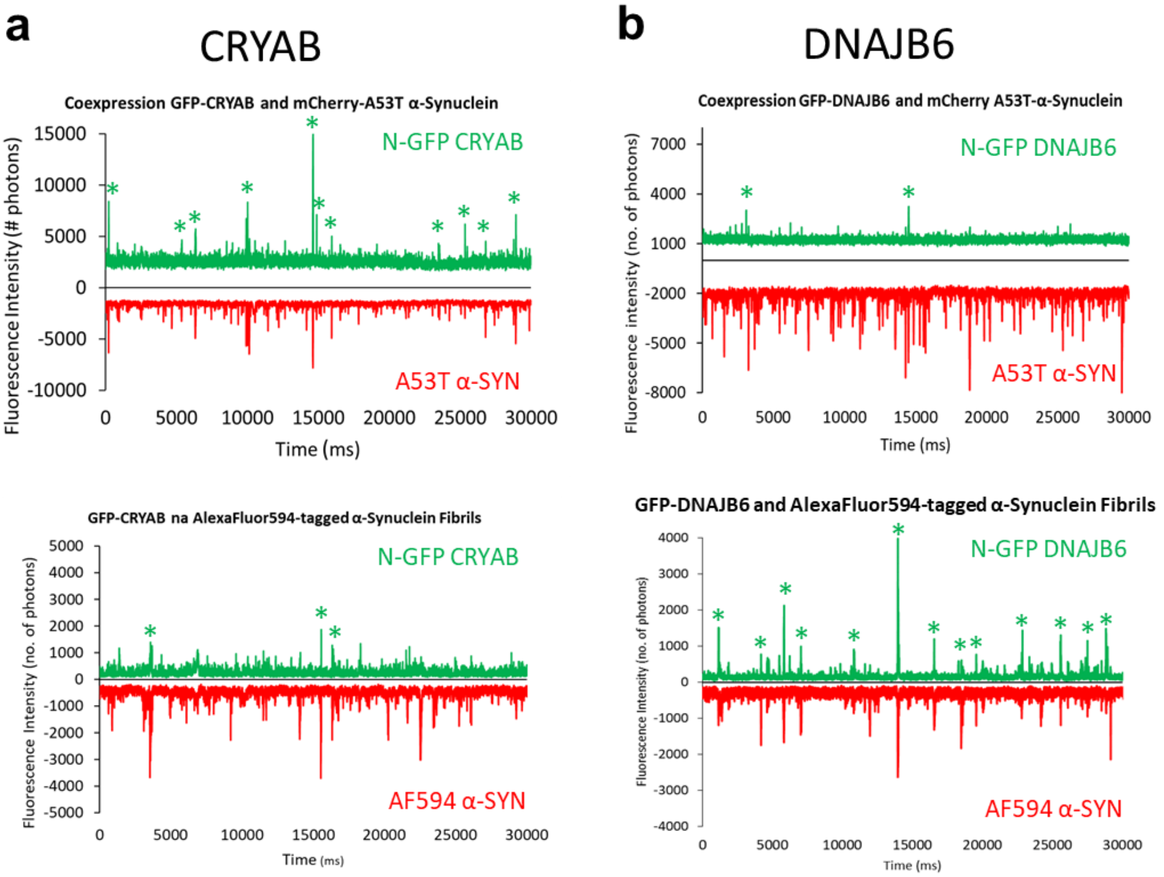

Figure S11. Selectivity of interactions to A53T oligomers versus amyloid fibrils of α-Synuclein. **a** Upper panel: coexpression of the small heat-shock protein alpha-crystallin B (CRYAB) with misfolding mutant A53T, which forms oligomeric structures. Bottom Panel: co-translational expression of CRYAB in cell-free extract with purified amyloid fibrils of alpha-synuclein. **b** Similar setup for the HSP40 protein, DNAJB6 showing no binding to oligomeric A53T and binding to fibrils. Asterisks represent CRYAB or DNAJB6 events that are coincident with aggregates of synuclein.

SUPPLEMENTARY FIGURE 12:

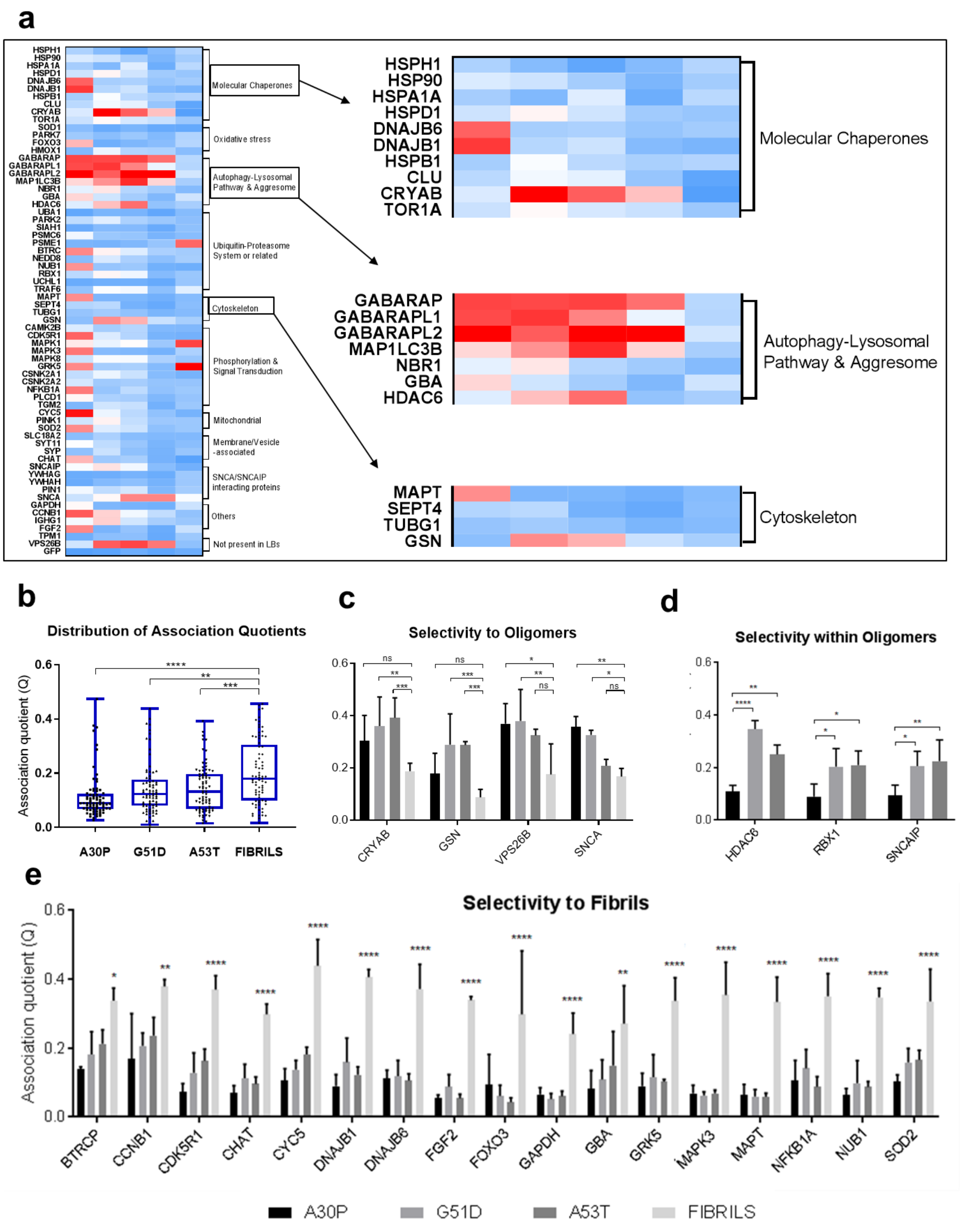

Figure S12. Selectivity in the recognition of α-SYN aggregates. a Heatmap of interactions highlighting specificities within protein families. **b** Q values were distributed for all the synuclein forms tested. **c** Selectivity to oligomers was defined as a statistically significant difference between one or more mutant forms of cell-free expressed synuclein and PFFs. **d** Few proteins showed a higher Q value for one of the mutant forms, as compared to the other two (selectivity within oligomers). **e** Selectivity to fibrils was defined as a statistically significant higher Q value for binding to fibrils as compared to the highest oligomer Q value of the same pairwise interaction. (Dunnett's multiple comparison test, **** p≤0.0001, *** p≤0.001, ** p≤0.01, * p≤0.05, n=3). Error bars show mean±SEM. This supplementary figure refers to **Figure 7**.

SUPPLEMENTARY FIGURE 13:

a

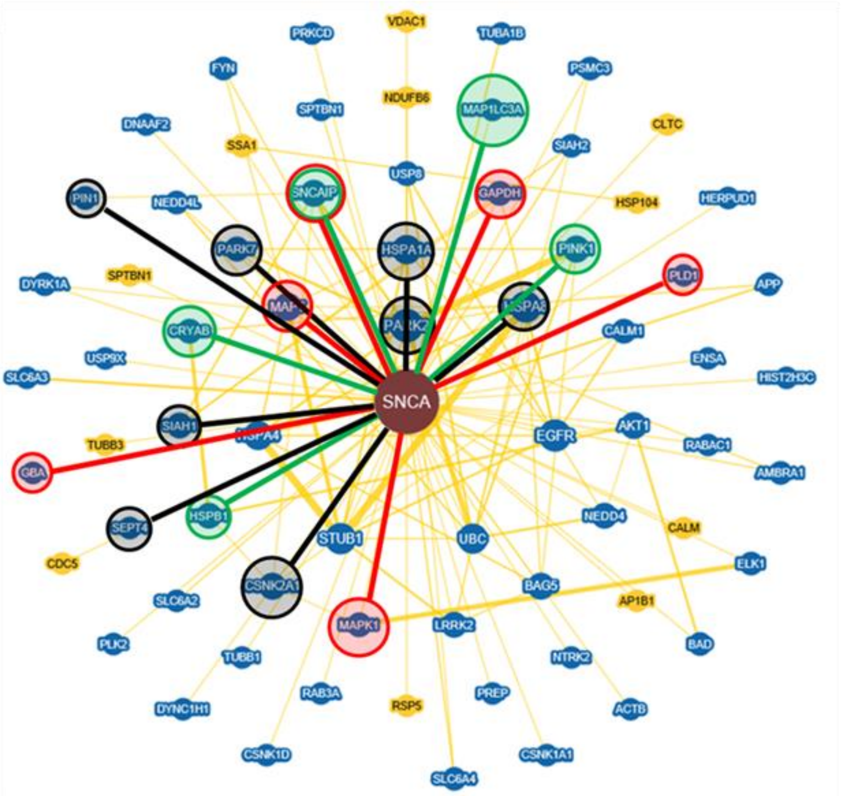

b

Previously documented interactors validated	Newly discovered interactions	Documented Interactors not validated	Interaction not documented and not Detected	Present in LBs but not tested	
				SNCA interactors	No PPI evidence
DNAJB1	FOXO3	HSP90	HSPH1	APP	CHIP
DNAJB6	GABARAP	HSPA1A	HSPD1	MAP1B	PCNT
CRYAB	GABARAPL2	HSPB1	CLU	LRRK2	CHGA
TOR1A	MAP1LC3B	PARK7	HMOX1	SQSTM1	AGRN
GABARAPL1	NBR1	PARK2	UBA1	MAP1S	HDAC4
GBA	HDAC6	SIAH1	PSMC6	MAP2	
MAPT	PSME1	TRAF6	NEDD8		
MAPK1	BTRC	SEPT4	TUBG1		
MAPK3	NUB1	CAMK2B	CSNK2A2		
GRK5	GSN	MAPK8	TGM2		
PLCD1	CDK5R1	CSNK2A1	SLC18A2		
CYCS	NFKBIA	SYP	SYT11		
PINK1	CHAT	YWHAG	YWHAH		
SOD2	CCNB1	PIN1	TPM1		
SNCAIP	IGHG1		GFP		
SNCA	FGF2				
GAPDH	VPS26B				
	RBX1				

Figure S13. Alpha-synuclein interactome in the LBs (comparison with published data). a Interactome of α-SYN, based on list of peer-reviewed published interactors, available in BIOGRID. **b** Interpretative table for the interactome of α-SYN. Highlighted interactions in insets A and B: red – interacted preferentially with fibrillar α; green – oligomeric mutant α-SYN (A30P, G51D or A53T); blue – monomeric WT α-SYN; black – no interaction was detected in this study; grey – previously described as present in LBs but not tested in this study - the high molecular weight of these proteins made them unsuitable for cell-free expression in Leishmania tarentolae. This supplementary figure refers to **Figure 7**.

SUPPLEMENTARY FIGURE 14:

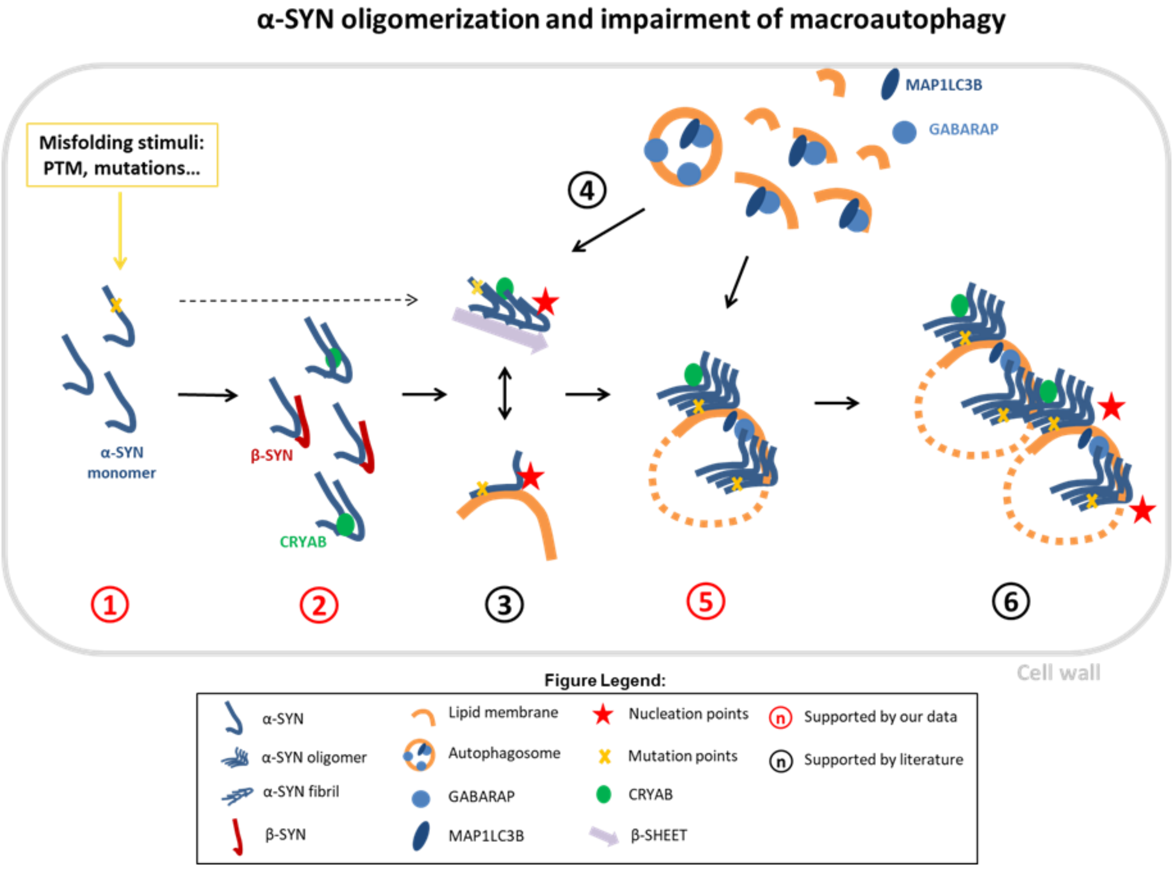

Figure S14. Proposed model of early α-Syn aggregation and Lewy Body formation.
1- Mutations and PTMs of the α-SYN molecule enhance its aggregation propensity. **2-** β-SYN and αβ-crystallin are chaperones of α-SYN that recognize early partially folded intermediates/aggregates, however faster-aggregating forms of α-SYN 'escape' proteostasis (dashed arrow). **3-** Membrane-bound oligomers and β-sheet fibrils coexist during the aggregation cascade of α-SYN and constitute nucleation points for further conversion and/or elongation. **4-** α-SYN aggregates co-diffuse in vitro with ATG8 proteins. **5 –** Co-diffusion of α-SYN oligomers with ATG8 proteins leads to growth of aggregates. **6-** Further growth and recruitment of aggregates potentially impairs autophagosome assembly.

SUPPLEMENTARY FIGURE 15

cLIR: [DE][DEST][WFY][DELIV]X[ILV]
xLIR: [ADEFGLPRSK][DEGMSTV][WFY][DEILQTV][ADEFHIKLMPSTV][ILV]

GENE	ID	Family	Motif (i)	Start	End	Pattern	PSSM (ii)	LIR in IDR (iii)	cLIR + IDR	xLIR+IDR+P>13	xLIR+IDR|P>13	OVERALL
BTRC	Q9Y297	Ubiquitin	xLIR	529	534	K V W D L V	13	No				
			xLIR	564	569	D E F Q I V	14	No				
CAMK2B	Q13554	Kinase	xLIR	88	93	L V F D L V	5	No				
			xLIR	236	241	P E W D T V	19	No				
CCNB1	P14635	Mitosis	xLIR	332	337	L D Y D M V	16	No				
CSNK2A1	P68400	Kinase	xLIR	10	15	R V Y T D V	7	No				
			xLIR	37	42	D D Y Q L V	17	No				
			xLIR	52	57	E V F E A I	8	No				
			xLIR	111	116	L V F E H V	5	No				
CSNK2A2	P19784	Kinase	xLIR	38	43	D D Y Q L V	17	No				
			xLIR	53	58	E V F E A I	8	No				
GSN	P06396	Actin binding	xLIR/cLIR	472	477	D S Y I I L	15	Yes				*
			cLIR	702	707	D T W D Q V	19	Yes				
HSP90AB	P08238	Chaperone	xLIR	506	511	R G F E V V	12	No				
HSPD1	P10809	Chaperone	xLIR	126	131	E G F E K I	12	No				
HSPH1	Q92598	Chaperone	xLIR	87	92	L S Y D L V	10	No				
			xLIR	714	719	K M F E E L	12	No				
NBR1	Q14596	autophagosome	xLIR	561	566	L S F E L L	10	Yes				
			xLIR/cLIR	730	735	E D Y I I I	17	Yes				*
NFKB1A	P25963	transcription factor	xLIR/cLIR	75	80	D S F L H L	13	Yes				*
PIN1	Q13526	isomerase	xLIR/cLIR	101	106	E D F E S L	16	No				
PLCD1	P51178	lipase	xLIR	5	10	R D F L T L	16	No				
PSMC6	P62333	proteasome	xLIR	265	270	D G F D T L	15	No				
SLC18A2	Q05940	transport	xLIR	73	78	D S F Q S I	13	No				
SNCAIP	Q9Y6H5	interacting partner	xLIR	4	9	P E Y L D L	12	No				
			xLIR	166	171	A S F T K V	9	No				
SYP	P08247	membrane association	xLIR	26	31	L G F V K V	9	No				
			xLIR	123	128	A T Y I F L	12	No				
SYT11	Q9BT88	membrane association	xLIR	299	304	L S Y Q P V	9	No				
TGM2	P21980	enzyme	xLIR	352	357	E G W Q A L	18	No				
TOR1A	O14656	enzyme	xLIR	126	131	S D Y V H L	17	No				
			xLIR	201	206	A M F I F L	11	No				
UBA1	P22314	ubiquitin	xLIR	157	162	S G F Q V V	11	No				
			cLIR	650	655	D E F E G L	13	No				
VPS26B	Q4G0F5	retromer	xLIR	107	112	F E F T H V	11	No				
MAP1B	P46821	cytoskeleton	xLIR	252	257	G G F L K L	10	No				
			xLIR	838	843	K D F E E L	16	Yes				
			xLIR	2355	2360	P V Y L D L	7	Yes				
SQSTM1	Q13501	autophagosome	xLIR	336	341	D D W T H L	24	Yes				
			xLIR	296	301	D V F D D I	10	Yes				
MAP2	P11137	cytoskeleton	xLIR	1243	1248	G E Y D K L	13	Yes				
			xLIR/cLIR	1289	1294	D D F I T V	17	Yes				
								Accuracy (%)	57.4	70.2	78.7	
								Sensitivity (%)	29.6	55.6	**96.3**	
								Specificity (%)	**95**	**90**	55	
								Balanced accuracy (%)	62.3	72.8	75.7	

Figure S15. Searching for functional LIR motifs in the LBs. a Canonical and extended LIR motifs (cLIR and xLIR respectively) were detected using a LIR search engine available online and compared against known LIR containing proteins (MAP1B, SQSTM1 and MAP2, bottom rows). This search engine detects the consensus sequences surrounding a core sequence of aromatic (W/F/Y) and hydrophobic aminoacids (I/L/V) – highlighted in yellow. Functional LIR motifs were selected using 3 factors (highlighted in red): **i)** presence of cLIR/xLIR; **ii)** overlap (more than 3 residues) with intrinsically disordered region (IDR), as detected by ANCHOR software; and **iii)** position specific scoring matrix (PSSM) >13. Using different combinations of these 3 factors (cLIR+A, xLIR+IDR+PSSM>13 and xLIR+IDR or PSSM>13), three functional LIR containing proteins were detected with high confidence (green in 'OVERALL' column), based on selectivity and specificity of each test: GSN, NBR1 and NFKBIA. Other proteins that could contain functional LIR motifs are represented in blue (however this is unclear due to low specificity). **b, c** Proteins containing functional LIR motifs co-aggregated with α-SYN, which could have deleterious consequences for the overall autophagic flux.

Supplementary Note (Figure S15)

If we consider the hypothesis that pathological α-SYN aggregates could overwhelm autophagic fluxes by binding/clustering to autophagosomes, it is then also worth investigating which other proteins in the Lewy Bodies bind to these structures and potentially increase convolution. We have scanned the list of Lewy Body components for the presence of LIR motifs that are required for binding to autophagosomal (ATG8) proteins, using a bioinformatical consensus sequence search engine[94] (**Figure S15**). Kalvari and colleagues[75] have recently refined the LIR motif to an extended LIR (xLIR) that represents functional motifs (experimentally validated) with higher accuracy than the canonical LIR (cLIR). Accuracy for functional LIR detection can be further increased when used in conjunction with a position-specific-scoring-matrix (PSSM) that analyses the probabilistic multialignment fit. In addition, LIR motifs that overlap with an intrinsically disordered region, as detected by ANCHOR software are more likely to represent functional motifs. This is because LIR's are generally extended sequences that exhibit characteristics of conformational switches, i.e. they fold upon binding to autophagosomes. Using this method we have detected with high accuracy at least three LIR-containing proteins in our list of LB proteins: gelsolin (GSN), neighbour to BRCA1 (NBR1) and NF-kappa-β inhibitor alpha (NFKBIA). While NBR1 is known to be involved in delivery of protein aggregates to autophagosomes for degradation, GSN and NFKBIA have not been identified before as binders of ATG8 proteins and could be important regulators of autophagy.

Coincidently, specific α-SYN aggregates have been shown to co-diffuse with all these proteins in our PPI screen. Interactions between α-SYN aggregates and these LB components could, in principle, lead to generalized loss of function and increase the number of species that become trapped in insoluble inclusions, potentially amplifying the collapse into the non-functional protein clumps that are Lewy Bodies.
