# Supplementary material for: Selectivity of Lewy body protein interactions along the aggregation pathway of α-synuclein
Source: Commun Biol. 2021 Sep 23;4:1124. doi: 10.1038/s42003-021-02624-x (PMC8460662; doi:10.1038/s42003-021-02624-x)
Supplement: Supplementary file 3 — Description of Supplementary Files [file 42003_2021_2624_MOESM3_ESM.pdf]

Description of Additional Supplementary Files

File name: Supplementary Data

Description: Data used to generate charts, organized as follows:

- Tab 1 – Main Figure 2
- Tab 2 – Main Figure 3
- Tab 3 – Main Figure 4
- Tab 4 – Main Figure 5
- Tab 5 – Main Figure 6
- Tab 6 – Main Figure 7
