# Supplementary material for: Selectivity of Lewy body protein interactions along the aggregation pathway of α-synuclein
Source: Commun Biol. 2021 Sep 23;4:1124. doi: 10.1038/s42003-021-02624-x (PMC8460662; doi:10.1038/s42003-021-02624-x)
Supplement: Supplementary file 5 — Reporting Summary [file 42003_2021_2624_MOESM5_ESM.pdf]

nature research

Corresponding author(s): YANN GAMBIN, EMMA SIERECKI

Last updated by author(s): Jun 24, 2021

Reporting Summary

Nature Research wishes to improve the reproducibility of the work that we publish. This form provides structure for consistency and transparency in reporting. For further information on Nature Research policies, see our Editorial Policies and the Editorial Policy Checklist.

Statistics

For all statistical analyses, confirm that the following items are present in the figure legend, table legend, main text, or Methods section.

n/a	Confirmed	
☐	☒	The exact sample size (*n*) for each experimental group/condition, given as a discrete number and unit of measurement
☐	☒	A statement on whether measurements were taken from distinct samples or whether the same sample was measured repeatedly
☐	☒	The statistical test(s) used AND whether they are one- or two-sided<br>*Only common tests should be described solely by name; describe more complex techniques in the Methods section.*
☒	☐	A description of all covariates tested
☒	☐	A description of any assumptions or corrections, such as tests of normality and adjustment for multiple comparisons
☐	☒	A full description of the statistical parameters including central tendency (e.g. means) or other basic estimates (e.g. regression coefficient) AND variation (e.g. standard deviation) or associated estimates of uncertainty (e.g. confidence intervals)
☒	☐	For null hypothesis testing, the test statistic (e.g. *F*, *t*, *r*) with confidence intervals, effect sizes, degrees of freedom and *P* value noted<br>*Give P values as exact values whenever suitable.*
☒	☐	For Bayesian analysis, information on the choice of priors and Markov chain Monte Carlo settings
☒	☐	For hierarchical and complex designs, identification of the appropriate level for tests and full reporting of outcomes
☒	☐	Estimates of effect sizes (e.g. Cohen's *d*, Pearson's *r*), indicating how they were calculated

Our web collection on statistics for biologists contains articles on many of the points above.

Software and code

Policy information about availability of computer code

Data collection	Data collection did not use any specific new software for acquisition
Data analysis	Codes used in this study were published previously and described in the Methods section

For manuscripts utilizing custom algorithms or software that are central to the research but not yet described in published literature, software must be made available to editors and reviewers. We strongly encourage code deposition in a community repository (e.g. GitHub). See the Nature Research guidelines for submitting code & software for further information.

Data

Policy information about availability of data

All manuscripts must include a data availability statement. This statement should provide the following information, where applicable:

- Accession codes, unique identifiers, or web links for publicly available datasets
- A list of figures that have associated raw data
- A description of any restrictions on data availability

The source data for graphs and charts is available as Supplementary data 1 and any remaining information can be obtained from the corresponding author upon reasonable request

Field-specific reporting

Life sciences study design

All studies must disclose on these points even when the disclosure is negative.

Sample size	In our in-vitro experiments, binding is calculated over hundreds to thousands of individual proteins. The duration of the acquisition and concentrations of the proteins used were calibrated to yield sufficient number of binding/co-aggregation events. In the case of co-aggregation with cell-free expressed oligomers, the scanning well method was used to increase the number of events, as described in the text and in the supplementary information section.
Data exclusions	No data were excluded from the analysis
Replication	The in-vitro experiments described in this manuscript were performed with three or more biological replicates, ie separate expressions with different cell-free extracts. LTE extracts have demonstrated reproducibility in terms of expression levels and binding or co-aggregation behaviors. Multiple batches of cell-free lysates were tested to ensure that the results are reproducible.
Randomization	Randomization is not relevant for this study, as individual experiments were performed and analysed in the exact same manner.
Blinding	In this large screen of interactions, blinding was not performed but experiments were performed in an agnostic fashion, without bias for any particular interactor.

Reporting for specific materials, systems and methods

We require information from authors about some types of materials, experimental systems and methods used in many studies. Here, indicate whether each material, system or method listed is relevant to your study. If you are not sure if a list item applies to your research, read the appropriate section before selecting a response.

Materials & experimental systems

n/a	Involved in the study	
☒	☐	Antibodies
☒	☐	Eukaryotic cell lines
☒	☐	Palaeontology and archaeology
☒	☐	Animals and other organisms
☒	☐	Human research participants
☒	☐	Clinical data
☒	☐	Dual use research of concern

Methods

n/a	Involved in the study	
☒	☐	ChIP-seq
☒	☐	Flow cytometry
☒	☐	MRI-based neuroimaging
